# Supplementary material for: Radiotherapy Upregulates the Expression of Membrane-Bound Negative Complement Regulator Proteins on Tumor Cells and Limits Complement-Mediated Tumor Cell Lysis
Source: Cancers (Basel). 2025 Jul 18;17(14):2383. doi: 10.3390/cancers17142383 (PMC12294122; doi:10.3390/cancers17142383)
Supplement: Supplementary file 1 [file cancers-17-02383-s001.zip › cancers-3726995-supplementary figures.pdf]

Supplementary Materials:

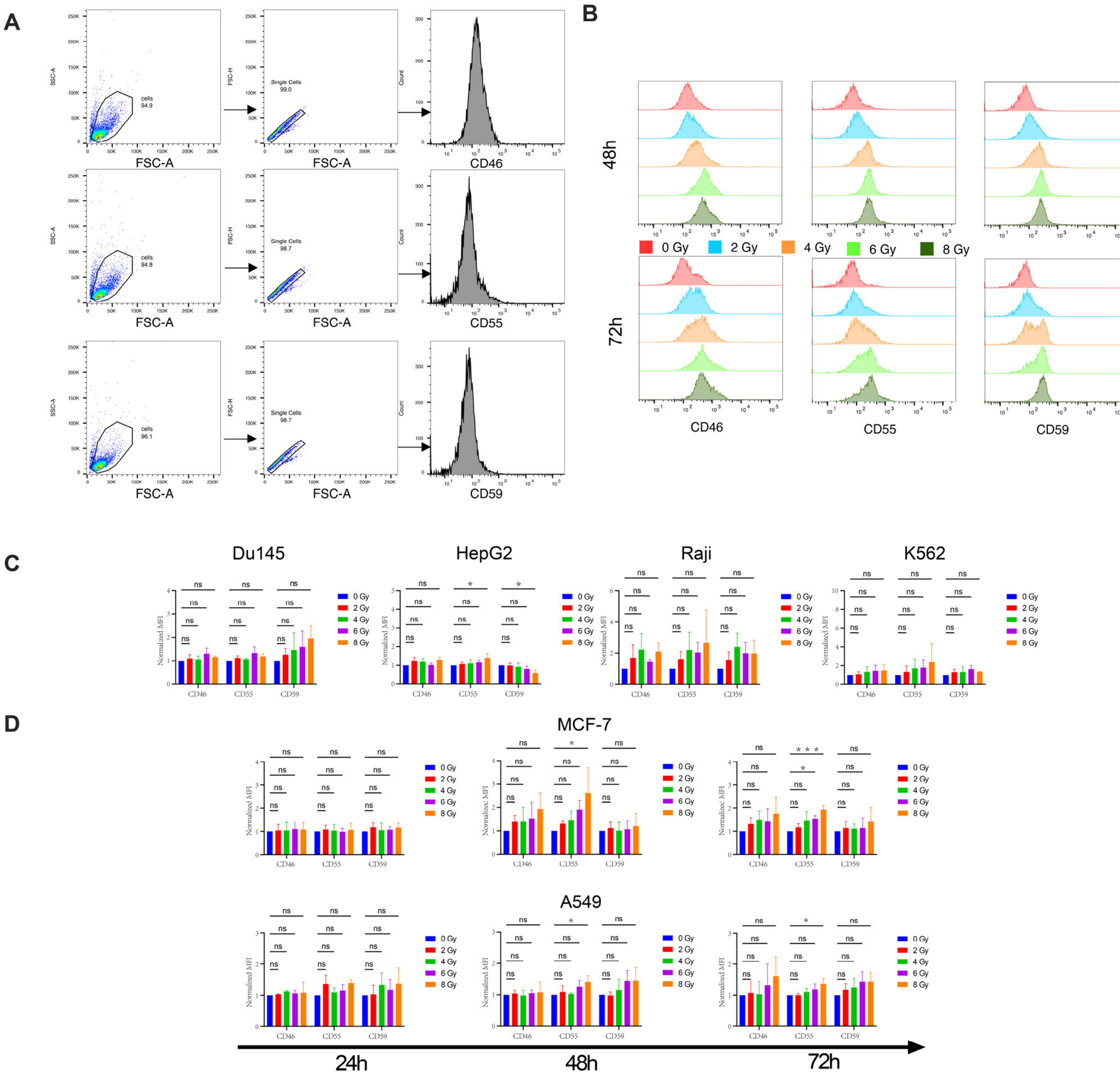

Figure S1. Impacts of radiotherapy (RT) on the expression of membranous complement regulatory proteins (mCRPs) on other different human tumor cells at different timepoints after RT. (A) The representative gating strategies to analyze the median fluorescence intensity (MFI) of each mCRP marker and the offset histograms of corresponding mCRPs of cell line Ramos at 48 hours and 72 hours after RT (B). RT upregulates the expression of mCRPs on other different human tumor cells 24 hours after irradiation(C). Impact of RT on the expression of mCRPs in breast cancer cell line MCF-7 and lung cancer cell line A549 at different time points (D). Different cancer cell lines were treated with different radiation doses as indicated and the MFI was analyzed by flow cytometry. Data was analyzed by one-way ANOVA with subsequent Dunnett's multiple comparisons testing; normalized MFI were calculated by dividing by the values of 0 Gy group. Data is given as mean ± SEM of 2-3 independent experiments per cell line. ns, not significant; *P < 0.05; **P < 0.01; ***P < 0.001.

RT: radiotherapy; mCRPs: membranous complement regulatory proteins; MFI: median fluorescence intensity.

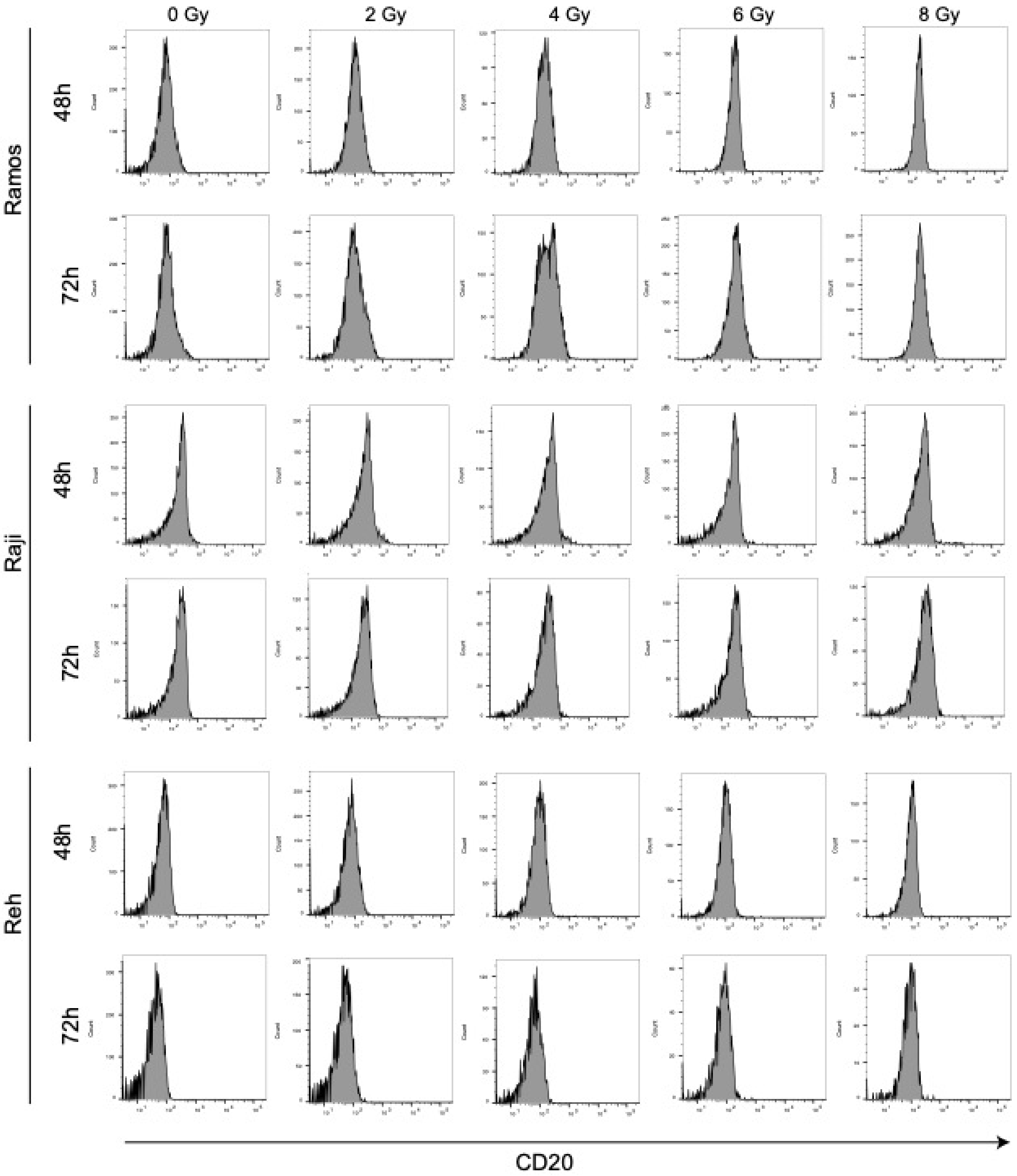

Figure S2. Representative histogram plots of CD20 expression in different lymphoma and leukemic cell lines. Cells were irradiated with 0 Gy, 2 Gy, 4 Gy, 6 Gy, and 8 Gy and cultured for 48 hours and 72 hours after irradiation. Cells were harvested and stained with anti-CD20 antibody conjugated with a fluorophore, and flow cytometry was used to detect the median fluorescence intensity of CD20 after gating the singlets.

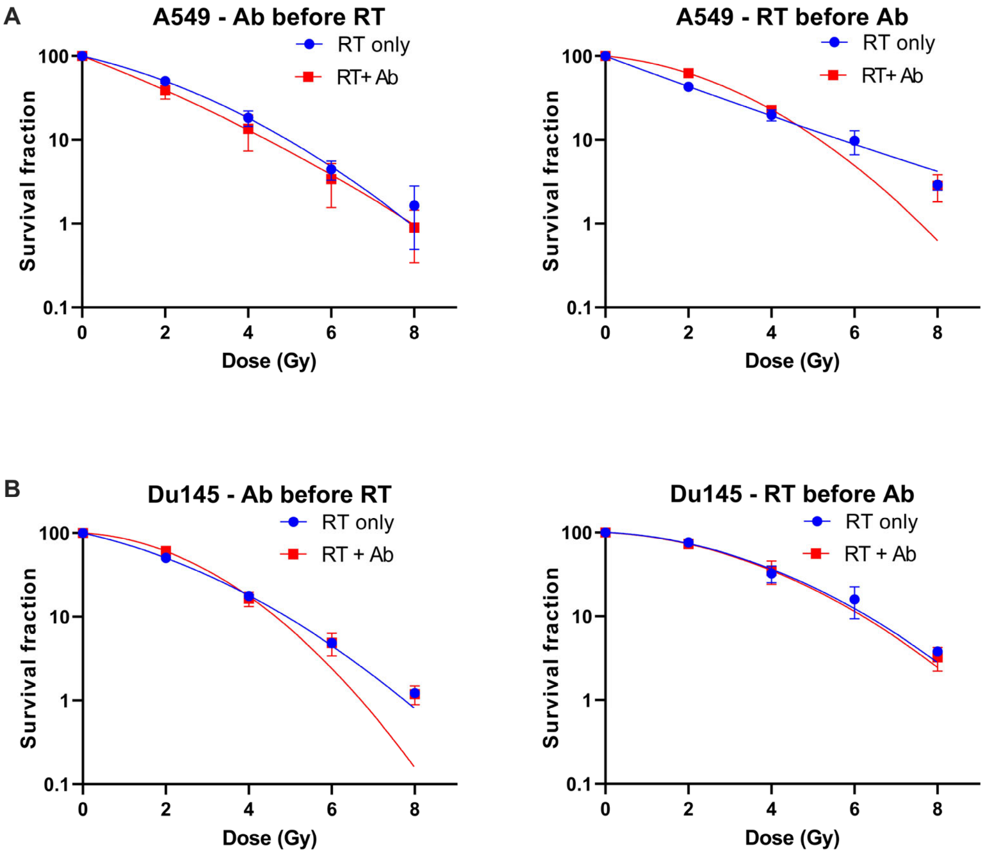

Figure S3. Complement activation does not significantly alter the intrinsic radiosensitivity of solid tumors. Lung cancer cell lines A549 (A) and prostate cancer cell line Du145 (B) were treated with complement-activating antibodies (CAB) and subsequent human serum (HS) either pre- or post-irradiation with different doses. Survival fraction is shown as mean of percentage ± SEM, survival curves were fitted with non-linear regression according to the linear-quadratic model.

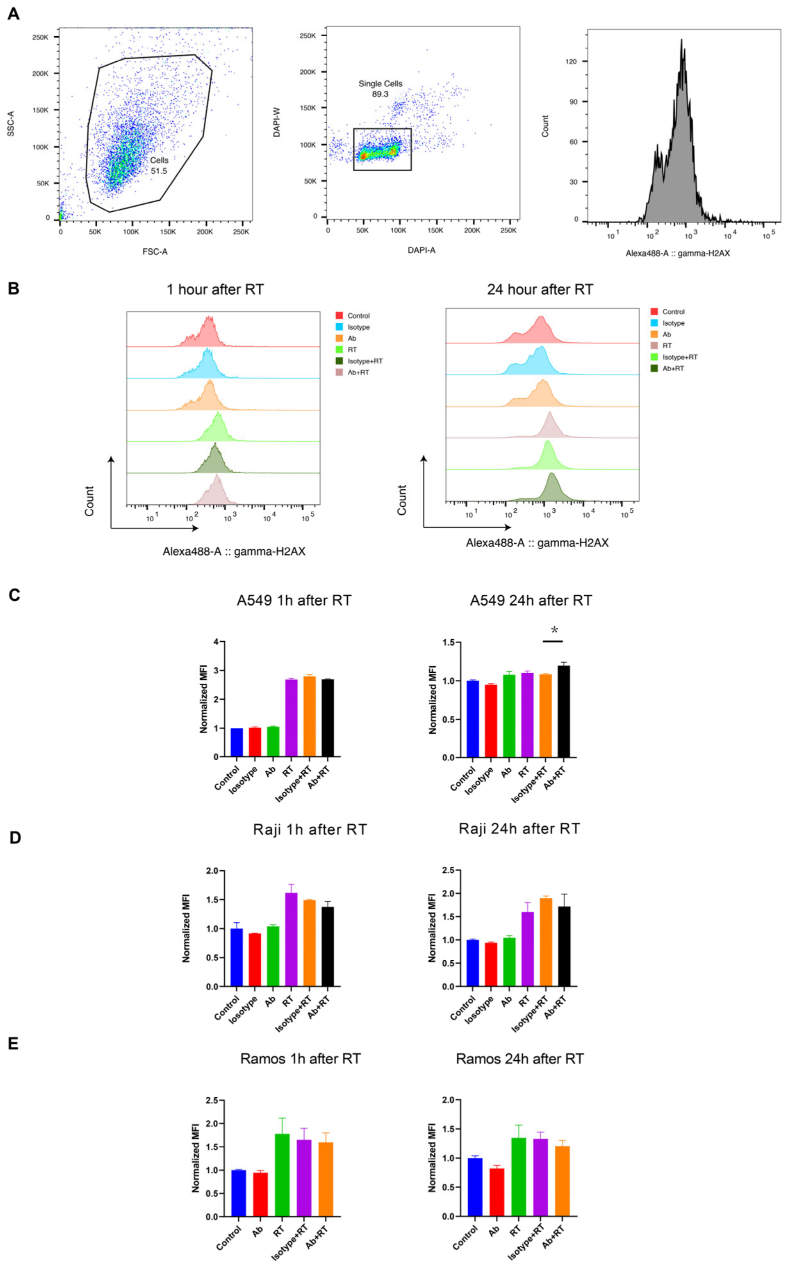

Figure S4. mCRPs blockade does not significantly alter DNA-damage repair kinetics after radiation in cancer cells. Human cancer cells were irradiated with a dose of 4 Gy, treated with mCRPs-blocking antibodies or left untreated (control). DNA-double strand breaks induction and repair kinetics were assessed via flow cytometry based γH2AX-detection 1 hour and 24 hours after radiotherapy (RT). Median fluorescence intensity (MFI) values were normalized towards the respective unirradiated control group. (A) Gating strategy, representative count histograms of γH2AX at 1 hour and 24 hours after RT in Raji cell line (B), and statistical bar charts of normalized MFI values from lung cancer cell line A549 (C) and lymphoma cell lines Raji (D) and Ramos (E) were shown. Data is given as mean values ± SEM of 2-3 independent experiments per cell line. Only statistically significant findings were indicated; *P < 0.05; **P < 0.01; ***P < 0.001 (two-tailed unpaired Student's t-test). mCRP: membrane-bound complement regulatory proteins; RT: radiotherapy.
